# Patient and healthcare professional perspectives on the Practical Guide to Implementing PROMs in Gender-Affirming Care (PG-PROM-GAC): analysis of open-ended responses from patients and healthcare professionals

**DOI:** 10.1136/bmjoq-2023-002721

**Published:** 2024-04-03

**Authors:** Rakhshan Kamran, Liam Jackman, Anna Laws, Melissa Stepney, Conrad Harrison, Abhilash Jain, Jeremy Rodrigues

**Affiliations:** 1 Nuffield Department of Orthopaedics, Rheumatology and Musculoskeletal Sciences, University of Oxford, Oxford, UK; 2 Temerty Faculty of Medicine, University of Toronto, Toronto, Ontario, Canada; 3 Northern Region Gender Dysphoria Service, Cumbria Northumberland Tyne and Wear NHS Foundation Trust, Newcastle upon Tyne, UK; 4 Department of Psychiatry, University of Oxford, Oxford, UK; 5 Department of Plastic Surgery, Buckinghamshire Healthcare NHS Trust, Amersham, UK; 6 Warwick Clinical Trials Unit, University of Warwick, Coventry, UK

**Keywords:** Implementation science, Healthcare quality improvement, Health services research, Patient Reported Outcome Measures

## Abstract

**Importance:**

Several international calls have been made for evidence-based patient-reported outcome measure (PROM) implementation for gender-affirming care. The Practical Guide to Implementing PROMs in Gender-Affirming Care (PG-PROM-GAC) is a resource which can help guide PROM implementation efforts, developed using a three-phase participatory research approach with transgender and gender-diverse (TGD) patients and gender-affirming healthcare professionals. However, thoughts and perspectives from TGD patients and gender-affirming healthcare professionals on the PG-PROM-GAC need to be investigated.

**Objective:**

Investigate patient and healthcare professional perspectives on the PG-PROM-GAC through analysis of open-ended survey results.

**Design:**

Qualitative study analysing open-ended responses from TGD patients and gender-affirming healthcare professionals.

**Setting:**

Participants were recruited from a UK National Health System (NHS) gender clinic.

**Participants:**

Patients receiving care at an NHS gender clinic and healthcare professionals working at an NHS gender clinic were eligible for participation. Eligible participants were invited to participate in this study via email.

**Intervention:**

Participants were sent an open-ended survey to collect responses on the PG-PROM-GAC.

**Main outcome(s) and measure(s):**

Data were thematically analysed by two independent researchers and interpreted following guidance from established methods in implementation science.

**Results:**

A total of 64 TGD patients and 9 gender-affirming healthcare professionals responded to the open-ended survey (mean (SD) age: 35 (16) and 48 (8), respectively). Four main themes emerged from the data: overall opinions and support for the PG-PROM-GAC, presentation of the PG-PROM-GAC, impact of gender clinic resources on PROM implementation and impact of PROM selection on implementation. Data were used to iterate the PG-PROM-GAC in response to participant feedback.

**Conclusions and relevance:**

The PG-PROM-GAC is an acceptable and feasible resource that can be used by clinicians, researchers and policymakers to guide PROM implementation for gender-affirming care settings, helping to align gender-affirming care with patient needs.

**WHAT IS ALREADY KNOWN ON THIS TOPIC**

- The Practical Guide to Implementing PROMs in Gender-Affirming Care (PG-PROM-GAC) is a resource which can help implement patient-reported outcome measures (PROMs) in gender-affirming care. However, thoughts and feedback from patients and healthcare professionals on the PG-PROM-GAC are currently unknown.

**WHAT THIS STUDY ADDS**

- This qualitative study analysing open-ended responses demonstrates that transgender and gender-diverse (TGD) patients and gender-affirming healthcare professionals viewed the PG-PROM-GAC as a thoughtfully constructed and needed resource for gender-affirming care. Feedback was also used to iterate the PG-PROM-GAC to become more user-friendly.

**HOW THIS STUDY MIGHT AFFECT RESEARCH, PRACTICE OR POLICY**

- The iterated PG-PROM-GAC is a resource which can help implement PROMs for gender-affirming care, and improve gender-affirming healthcare quality and delivery.

## Introduction

Gender-affirming care is a key clinical area which can benefit from systematic and evidence-based implementation of patient-reported outcome measures (PROMs).^1^ PROMs are self-report questionnaires quantifying how patients feel and function.^2^ Implementation of PROMs for gender-affirming care can help to ensure care is provided in line with international clinical guidelines,^3^ align care with patient needs, improve patient-centredness, and challenge bias in treatment or poor patient care where appropriate.^1 4^ However, in numerous clinical settings, the benefits of PROMs are not realised to their full potential due to implementation challenges.^5^ Evidence-based PROM implementation strategies offer a solution to maximise PROM implementation.^6^ Improving gender-affirming care quality is an international healthcare priority, and implementing PROMs can help to achieve this.^1 3^

The Practical Guide to Implementing PROMs in Gender-Affirming Care (PG-PROM-GAC) is a resource which can help clinicians, researchers and policymakers implement PROMs more effectively and consistently for their gender-affirming care setting.^7^ Over 200 different PROMs for adult gender-affirming care and 38 different PROMs for paediatric gender-affirming care have been identified which have the potential to be implemented into clinical practice.^1 8^ This large number of existing PROMs contributes to potential redundancy and complexity with PROM implementation as guidance is lacking on the best PROMs to implement for gender-affirming care. Local service improvement initiatives can be used to help guide selection of the best PROMs to use relevant to local settings. The PG-PROM-GAC contains two tables with sections which can help guide PROM implementation: one section focuses on patient-relevant strategies, and another section focuses on healthcare professional (HCP)-relevant strategies.^7^ The PG-PROM-GAC is also being used by the leads of a National Health System (NHS) England workstream for PROMs in gender clinics to help implement PROMs in NHS gender clinics. The PG-PROM-GAC has been developed over the past 3 years and is an output of previously reported projects from a University of Oxford doctorate including the following:

- A systematic review of 286 international articles (representing 85 395 transgender and gender-diverse (TGD) patients).^1^
- A qualitative study representing 14 TGD patients and 10 interdisciplinary gender-affirming HCPs.^9^
- Iteration and refinement with seven TGD patients and a gender-affirming HCP.^7^
- Measurement of acceptability, appropriateness and feasibility of the guide through a cross-sectional study using validated implementation surveys, based on 132 TGD patients and 13 gender-affirming HCPs.^10^
- Future research will encompass real-world deployment of the PG-PROM-GAC and measuring its effectiveness when used to implement PROMs at an NHS gender clinic.

While quantitative data supporting the acceptability, appropriateness and feasibility of the PG-PROM-GAC exist, why patients and HCPs chose to score this way is not clear.^10^ Measurement of patient perspectives is essential in ensuring implementation strategies are relevant and meaningful to patients.^11^ The aim of this study was to address this gap through investigating patient and HCP perspectives on the PG-PROM-GAC through analysis of open-ended survey results. This is an independent study to build on previous PROM implementation work and fill a literature gap for gender-affirming care.

## Methods

### Patient and public involvement

Seven patient and public members from the TGD community were involved with this research. This patient and public involvement group confirmed relevance and importance of this research, and confirmed the applicability and relevance of findings. Patient and public members were recruited through local and national TGD charity organisations and community support groups.

### Reporting

The Standards for Reporting Qualitative Research guideline^12^ was followed for this article.

### Approach and research paradigm

We used an interpretive description approach as this focuses on generating knowledge relevant to clinical contexts and applied health disciplines.^13–15^ Interpretive description approaches have been previously applied for studies on PROM development,^16^ and have relevance to this study which aims to generate knowledge on participant thoughts and perspectives on the PG-PROM-GAC (online supplemental appendix 1). We used thematic analysis to analyse the data and normalisation process theory (NPT)^17 18^ as a guiding theory as it is a key implementation science theory which aims to understand how an innovation (eg, PROMs) may become routinised in practice. NPT is a middle-range theory which was used to support the development of the research question and focus, aid with interpretation of the results (keeping interpretation focused on implementation concepts), and to guide and structure conclusions and recommendations.^17^

10.1136/bmjoq-2023-002721.supp1 Supplementary data

### Researcher characteristics and reflexivity

Data were analysed by a doctoral candidate formally trained in qualitative research and a research specialist in patient-reported outcomes (RK), and a master’s candidate with prior experience in PROM implementation and qualitative research (LJ). A relationship was not established with participants prior to this study. The researchers involved with data analysis were involved in designing the research question, qualitative approach, methods and reporting results. To aid reflexivity, memos and notes were drafted by researchers during data analysis to build awareness of their own positionality and how this affects the research process.

### Context

The clinical context for this study is gender-affirming care. Gender-affirming care comprises of a broad range of psychosocial, hormonal and surgical treatments to help gender dysphoria; however, it is not uniformly available across the globe and for young TGD people in particular, has been increasingly subject to discriminatory legislation and medical mandates to limit gender-affirming treatment. Adult participants (aged 18 years or older) for this current study were recruited from the UK, where trans people experience a number of barriers to accessing gender-affirming healthcare.^19^

### Sampling strategy

Participants at a gender clinic (n=1859) and HCPs at a gender clinic (n=32) were contacted via email with an invitation to participate in this study. The email outlined that participation involved reviewing the PG-PROM-GAC, a short introduction to the PG-PROM-GAC and how it was developed with service user input, and inviting participant feedback through three open-ended questions: (1) Are there any thoughts or comments you would like to share about the patient-relevant PROM Implementation Strategy Guide?; (2) Are there any thoughts or comments you would like to share about the HCP-relevant PROM Implementation Strategy Guide?; (3) Are there any final thoughts or comments you would like to share? The rationale for participants to provide comments via an open-ended survey was to capture a large number of perspectives from individuals representing a broad range of gender identities.^20^ A reminder email was sent 1 week after the initial participant recruitment email. Beyond the information included in the introductory email, participants had not been exposed to PROMs in the clinic before.

A total of 64 TGD patients and 9 HCPs responded to the open-ended survey. The mean (SD) age of TGD participants is: 35 (16) with a range of ages between 18 and 71 years. TGD participants’ self-reported gender were mostly female (29, 45%) or male (16, 25%); and female (26, 41%) or male (37, 58%) sex assigned at birth. Most TGD participants were white (61, 95%) and British (52, 81%). For the HCP sample, participants had a mean (SD) age of 48 (8) with a range of ages between 29 and 57 years. Most HCP participants were female gender (7, 78%) and female (6, 67%) or male (3, 33%) sex assigned at birth. Most HCP participants were white (8, 89%) and British (8, 89%). Table 1 displays demographic information for study participants.

**Table 1:** Demographic information of study sample

Demographic information	Frequency (%)
**TGD patient characteristics (n=64)**	
Age, mean (SD) (n=63)	35 (16)
Gender*	
Female	29 (45)
Genderqueer	1 (2)
Genderfluid	1 (2)
Male	16 (25)
Non-binary	2 (3)
Queer	1 (2)
Trans female	4 (6)
Trans femme	1 (2)
Trans male	3 (5)
Trans masculine	3 (5)
Transgender	1 (2)
NR	2 (3)
Sex assigned at birth*	
Female	26 (41)
Male	37 (58)
NR	1 (2)
Race†	
Mixed—European	1 (2)
White	61 (95)
NR	2 (3)
Ethnicity†	
American	1 (2)
British	52 (81)
British and Irish	2 (3)
British and European	1 (2)
Celtic	1 (2)
Irish	3 (5)
Mixed European	1 (2)
Welsh and British	1 (2)
NR	2 (3)
**Healthcare professional characteristics (n=9)**	
Age, mean (SD) (n=9)	48 (8)
Gender*	
Female	7 (78)
Genderqueer femme	1 (11)
Male	1 (11)
Sex assigned at birth*	
Female	6 (67)
Male	3 (33)
Race†	
Mixed—white and Asian	1 (11)
White	8 (89)
Ethnicity†	
British	8 (89)
European	1 (11)

*Gender was measured using the two-step method via open-ended response where participants were first asked their gender and then their sex assigned at birth.

†Race and ethnicity information were collected via open-ended responses to allow for self-identification rather than forcing participant responses into predetermined categories which may not address how participants self-identify.

NR, not reported; TGD, transgender and gender-diverse.

### Data collection methods, instruments and technologies

Data collected included demographic information of study participants (age, gender, sex assigned at birth) and responses to three open-ended questions. The data collection instrument (online survey) was pilot tested with a patient and public involvement group and a HCP. Data collection began on Thursday 7 September 2023 until Thursday 21 September 2023. Data were collected on Microsoft Forms. Data were analysed iteratively from 7 September 2023 to 27 November 2023.

### Data processing

Data were managed and coded on Microsoft Excel (V.16.69) and stored on an encrypted cloud server based at the University of Oxford to ensure data security. Data were anonymised with participants being assigned a participant ID on Microsoft Forms during the data collection phase.

### Data analysis

Data were thematically analysed with coding of open-ended responses occurring independently and in duplicate.^20^ In line with current recommendations for qualitative analysis of open-ended survey responses,^20^ the data for this study were analysed as one cohesive dataset with analytical patterns developed across the entire dataset rather than analysing responses to each question separately. The rationale for this is that although a specific question may direct participants to share a particular perspective, relevant perspectives to a particular question may be shared in responses to other questions as well.^20^ Regular debriefing meetings between researchers covering key concepts from data analysis were held to ensure rigour.^21^ NPT was used to help guide emerging interpretations, conclusions and recommendations from the data.^17^

### Techniques to enhance trustworthiness

Results were provided to participants to check for accuracy to ensure credibility and trustworthiness.^22^ Results were also provided to patient and public partners to ensure relevance and meaningfulness of study results to the TGD community. Service users provided input to improve the simplicity of language used and overall comprehensibility of questions.

## Results

**Figure 1:**
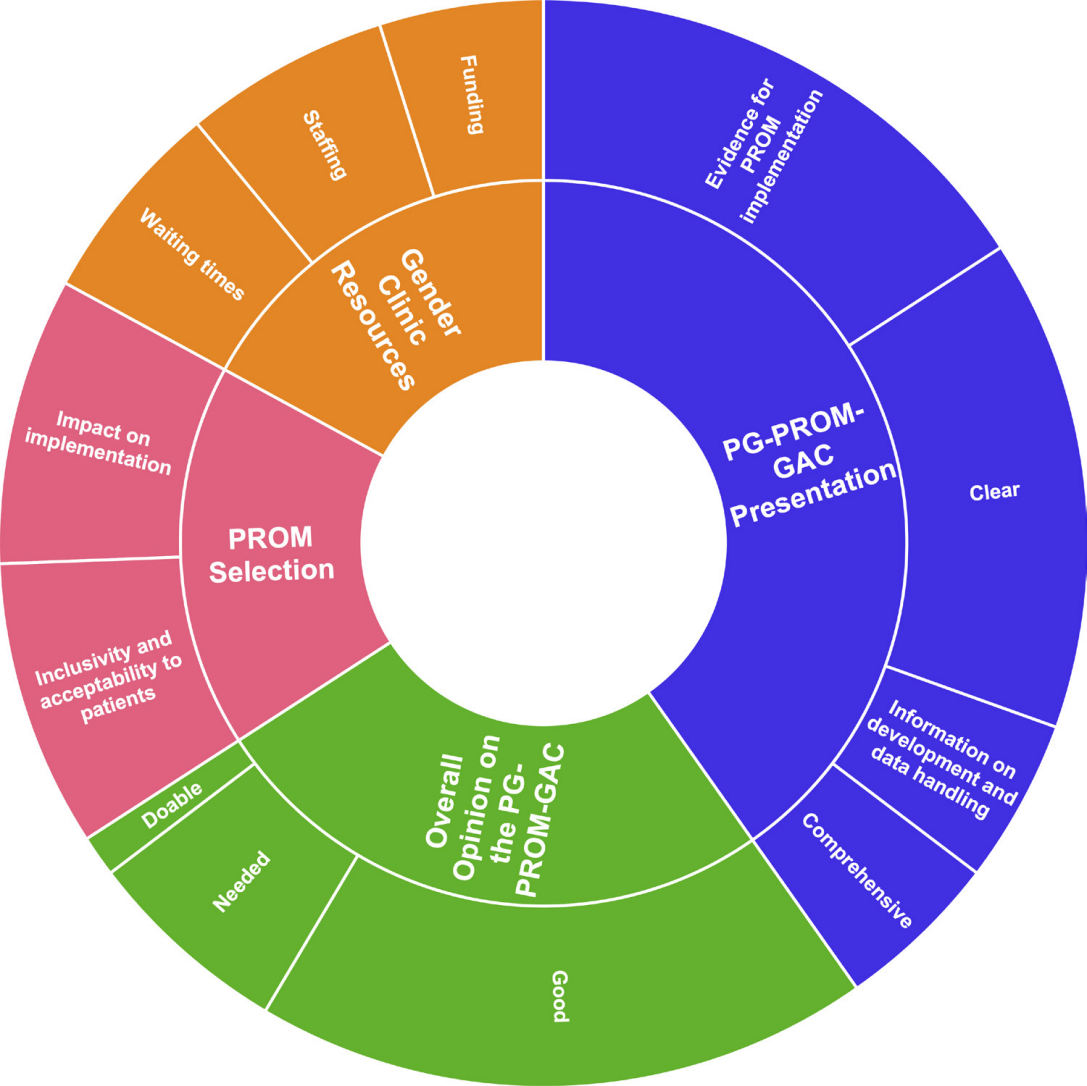
Sunburst chart representing frequency of codes. PG-PROM-GAC, Practical Guide to Implementing PROMs in Gender-Affirming Care; PROM, patient-reported outcome measure.

A total of four main themes relating to: overall opinion on the PG-PROM-GAC, presentation of the PG-PROM-GAC, impact of gender clinic resources on PROM implementation and impact of PROM selection on implementation were identified (figure 1). These themes were interconnected and comprised of positive comments about the PG-PROM-GAC as well as suggestions for iteration. Participants also highlighted specific strategies mentioned in the PG-PROM-GAC that they felt were highly important for PROM implementation. Below, each theme will be illustrated, and the linking of concepts is discussed.

### Overall opinion on the PG-PROM-GAC

Overall, TGD participants and gender-affirming HCPs supported the PG-PROM-GAC as a whole. Specifically, participants described how the PG-PROM-GAC was needed for gender-affirming care, with some saying they welcomed the PG-PROM-GAC, and that they agreed with the content in the PG-PROM-GAC.

The [PG-PROM-GAC] guide is what [gender-affirming care] patients need. (Female patient, aged 43 years)

I understand and agree with the [PG-PROM-GAC] guide. (Female patient, aged 22 years)

I support the [PG-PROM-GAC] initiative and welcome it. (Female patient, aged 59 years)

We need this [PG-PROM-GAC] in gender healthcare. (Genderqueer female HCP, aged 50 years)

I feel that the [PG-PROM-GAC] Guide has been written with a high level of consideration and empathy towards patients. (Non-binary patient, aged 30 years)

Most participants supported and welcomed the PG-PROM-GAC, specifically writing support for the content in the PG-PROM-GAC and how this can be used to improve gender-affirming healthcare.

I think this [PG-PROM-GAC] guide was a good idea on PROMs and helping people get better care that they need. (Transgender male patient, aged 29 years)

Some participants went into greater detail on specific strategies within the PG-PROM-GAC they felt were highly important. Specific strategies felt to be important from the PG-PROM-GAC (available in online supplemental appendix 1) by participants include PROM accessibility and timing of PROM administration:

I hope it is administrated to patients in an appropriate manner as gender clinic sessions for example can be very emotionally loaded for patients, so perhaps as a follow up email would give patients time to wind down and collect their thoughts beforehand. (Male patient, aged 21 years)

I think the idea of having different accessible versions is really good. I think if someone needs support then there needs to be an independent source that can help eg, peer support workers if they are not directly being asked about. (Genderqueer patient, aged 35 years)

This same participant (genderqueer patient, aged 35 years) also felt the strategy on sharing knowledge and information between clinics (see online supplemental appendix 1) should be undertaken with care and sensitivity. They had concerns about data sharing:

Sharing information between clinics seems really important in terms of sharing learning but it would be need to be very clear for patients whether they want that to happen. Which could prove problematic if it leads to a skew in the data that is shared. (Genderqueer patient, aged 35 years)

The view that data consent and security was important was shared by many participants. The importance of ensuring transparency of data handling was a shared theme among many participants and expanded beyond the strategy of sharing information between clinics to cover all aspects of general PROM implementation:

Explanation of how the data will be used, how it will be kept secure and not used against the patient or service at a later date is a key part of engaging patients. A lot of trans patients are suspicious of healthcare processes and how information can be used to gatekeep services and obstruct their treatment pathway. (Female patient, aged 41 years)

Overall, these comments suggest that the strategies of education around PROM implementation, ensuring and communicating information on data transparency, and confirming patient consent with who they would like PROM data to be shared with are highly important and should potentially be viewed as linked strategies rather than discrete entities.

### PG-PROM-GAC presentation

The presentation of the PG-PROM-GAC (eg, layout of text) was commonly discussed by participants. Participants discussed positive elements of the presentation of the PG-PROM-GAC. Positive comments for how the PG-PROM-GAC was presented included participants feeling that the PG-PROM-GAC was clear, comprehensive and covered various areas of relevance for PROM implementation in gender-affirming care.

I like that it [PG-PROM-GAC] has covered various areas of accessibility, information governance and education. (Female HCP, aged 29 years)

Participants mentioned that the benefits of PROMs are important to explain, specifically how PROMs may benefit patients and gender-affirming healthcare. Participants feeling that the information in the PG-PROM-GAC helped them understand the reasons for PROM implementation and how this could be helpful are illustrated below. This also links to the themes of trust with PROM implementation and engaging participants to complete PROMs, as highlighting PROM benefits may help to increase trust and engagement.

I think this [PG-PROM-GAC] is a good idea because being a trans person is difficult at times because of the insecurity that you weren’t born female/male and always comparing yourself to others. I think a PROM is a good thing to introduce to people because people need to know how trans people feel and how transitioning affects them either good or bad. (Female patient, aged 19 years)

However, not everyone felt this way, and some individuals disagreed that the PG-PROM-GAC was clear and comprehensive. Some participants expressed confusion with the use of acronyms, and others felt the guide could benefit with evidence around the rationale for PROM implementation and evidence for PROM benefits.

Whilst there’s some good content, it [PG-PROM-GAC] doesn’t explain or incentivise healthcare professionals to get involved or implement the recommendation. What’s in it for them? It should be putting forwards methods of delivering - good practice for example. Needs to be easy take on board and implement, at present this just looks like more work for them. (Patient)

A few strategies within the PG-PROM-GAC relate to this participant comment, specifically the patient-relevant strategy of having educational information about PROMs and the HCP-relevant strategy of developing educational material on PROMs. This comment emphasises that communication of these strategies alone may not be enough, and that the PG-PROM-GAC should be amended to include some educational information which may help to incentivise PROM implementation. Some participants provided feedback on additional information that the PG-PROM-GAC should cover, specifically additional information on how the PG-PROM-GAC was developed and how the input of service users and HCPs was used.

I would like to know how many trans service users have been involved in the creation of the [PG-PROM-GAC] guide as well as healthcare professionals. (Male patient, aged 25 years)

In addition to transparency with PROM data handling, this comment exemplifies the need for transparency with how the PG-PROM-GAC was developed, specifically how trans service users have been involved in the process. When including additional information in the PG-PROM-GAC, it is important that the information be presented in an engaging way. Some participants felt that the PG-PROM-GAC in its current state was too wordy.

I think the idea [PG-PROM-GAC] is a good one, however the document is very wordy and makes the process seem very complicated when it doesn’t need to be. (Female HCP, aged 44 years)

Presentation style is very poor, it [PG-PROM-GAC] appears as a wall of words. It really needs to engage the audience. (Patient)

These comments exemplify that when amending the PG-PROM-GAC to include additional information, care should be taken to present information in an engaging way. Strategies to improve presentation in the PG-PROM-GAC may include the use of graphics to reduce the document appearing as a ‘wall of words’, using colours to highlight key concepts within strategies and including alternative forms of communication (ie, videos) which may help to enhance presentation of the PG-PROM-GAC and engagement with participants who mentioned areas for improvement.

### Gender clinic resources

Participants frequently discussed the impact of healthcare resources on real-world deployment of the PG-PROM-GAC. Specifically, while participants spoke to the theme of supporting the PG-PROM-GAC (as was discussed in a previous section), many were unsure as to what using the PG-PROM-GAC would look like. Specific concerns participants had were around the limited resources in gender-affirming care, with participants discussing that gender clinics may have limited staffing, funding and a high volume of patients being seen and on the waiting list which could potentially impact use of the PG-PROM-GAC.

I feel like it [PG-PROM-GAC] is going to be a big change on already scarce resources, and will need more people power or a dedicated person to ensure this is done as it should be. (Female HCP, aged 45 years)

Some things may be tricky, like dedicated rooms and support from staff. (Female patient, aged 44 years)

Specific strategies in the PG-PROM-GAC which were mentioned as needing to be considered in relation to the resources a gender clinic has include having a dedicated and private space to complete PROMs, and identifying and preparing implementation champions to help with PROM implementation which may also include involving staff members. What these comments suggest is that viewing all strategies of the PG-PROM-GAC as mandatory to have in place may cause disengagement with the PG-PROM-GAC. These comments are also related to a strategy in the PG-PROM-GAC which highlights the importance of tailoring PROM implementation to local settings. Highlighting this information in a clearer way at the beginning of the PG-PROM-GAC, through including guidance on how the PG-PROM-GAC can be used, and that strategies should be considered within the local implementation context, may help to increase engagement.

### PROM selection

Some participants discussed the impact PROM selection may have on the PG-PROM-GAC. It was important for participants who discussed this concept to have a PROM implemented which is viewed as inclusive and acceptable for TGD patients.

[The] devil is in the detail of which PROM is selected, the nature and inclusivity of the selection process and the acceptability to patients. So, we need something in here about how we will use this to allow staff to reflect on their practice and provide evidence of their standard of care, we need something about how this might integrate with service development and improvement through audit and possibly research. We need to know how it will be resourced without taking resource away from patient care. (Male HCP, aged 57 years)

I understand why they are used but some PROMs are quite dehumanising. My experience of using this style of survey for 18 years or so is that the questions no longer hold any meaning. (Trans masculine patient, aged 32 years)

These comments link to the themes mentioned about trust and patient engagement, specifically trust in that PROM implementation can help to improve gender-affirming healthcare. These comments illustrate that some participants feel it is important that PROM implementation does not add to the burden of clinicians providing care, and that the benefits of PROMs to patients need to be clear. A few participants also commented on the importance of selecting a PROM that is not overly burdensome to administer and score.

I think the detail of what is used will make or break this [PG-PROM-GAC]. If it [PROM] is 20 pages of tick boxes or a meaningful attempt to capture feedback. (Female HCP, aged 57 years)

Participants felt that patients would not feel engaged to complete PROMs if they were overly burdensome or viewed as unacceptable. This highlights an important concept relevant to the PG-PROM-GAC, that the PROM selected for use may impact the effectiveness of the listed implementation strategies.

## Discussion

This study presents the perspectives of 64 TGD patients and 9 gender-affirming HCPs on the PG-PROM-GAC. Key suggestions moving forward with the PG-PROM-GAC include selecting a PROM which patients find accessible and relevant to local gender-affirming care settings. This can be achieved through service improvement initiatives aiming to determine patient acceptability of a shortlist of PROMs which have been identified by staff in a gender-affirming care clinic.^23^ Second, additional information supporting the development of the PG-PROM-GAC can be presented when using the PG-PROM-GAC.^1 7 9 10^ Third, the PG-PROM-GAC can be adaptable and tailored to meet the needs based on local needs of gender-affirming care settings. This can be done through local service improvement initiatives using the voices of patients and HCPs for a specific gender-affirming care setting to refine the PG-PROM-GAC for their local context. The PG-PROM GAC is also developed to work alongside whichever PROM a gender-affirming care setting chooses to implement. The PG-PROM-GAC includes suggestions on PROM selection to consider participant burden, and including service user input for the specific gender-affirming care setting aiming to implement PROMs. Fourth, the strategies in the PG-PROM-GAC should be viewed as complementary to address key implementation concerns local gender-affirming care settings may have. Rather than viewing each strategy in the PG-PROM-GAC as a discrete entity, clinicians, researchers and policymakers should consider that using a combination of strategies together can potentially maximise effectiveness of PROM implementation. While the PG-PROM-GAC was developed following studies in gender-affirming care settings, and is an overall ready-to-use resource which can help to implement PROMs for gender-affirming care, it has potential generalisability to other specialist areas. Other specialist areas beyond gender-affirming care implemented in PROM implementation can use the PG-PROM-GAC to help guide their own implementation initiatives.

This study demonstrates overlap between what patients and HCPs discussed when giving feedback for the PG-PROM-GAC. Participants mentioned overall support for the PG-PROM-GAC and discussed that the PG-PROM-GAC was needed for gender-affirming healthcare, and thoughtfully constructed. However, an important consideration for participants was transparency with data handling, and the implications this can have on patient trust and engagement. This underscores the importance of the first patient-relevant strategy within the PG-PROM-GAC around education and the importance of the third HCP-relevant implementation strategy within the PG-PROM-GAC around staff responsibility for integrity with data processing and collection.

When comparing the results of this research with other clinical fields, there is some overlap. In a qualitative study of PROM implementation for general practitioner practice, it was found that evidence on PROM implementation benefits is important to communicate with end-users to enable uptake of implementation plans, in line with our findings.^24^ In a qualitative study of PROMs for oncology practices, it was found that having guidance for PROM implementation can improve uptake.^25^ This is in line with our findings demonstrating the need and usefulness of the PG-PROM-GAC to help guide PROM implementation. In a qualitative study of PROM implementation in rheumatology, a key finding was providing resources and programmes to help integrate PROMs as part of care.^26^ This finding is also in line with the theme of support for the PG-PROM-GAC which was found in this study, as well as the concepts of coherence/sense-making and collective action, covered under NPT.^17^ As there are several PROM implementation initiatives ongoing in diverse clinical areas, consolidation of initiatives may help to improve consistency of strategy design and sharing of knowledge and resources.

Regarding the theme of selecting a PROM to use alongside the PG-PROM-GAC, potential instruments which may be valuable to gender-affirming care settings include: the Gender Congruence and Life Satisfaction Scale,^27^ the Utrecht Gender Dysphoria Scale,^28^ and the Gender Identity/Gender Dysphoria Questionnaire for Adolescents and Adults.^29^ These PROMs have been developed and validated for gender-affirming care settings. Reducing the burden of PROMs can be done through techniques in computerised adaptive testing, which can make PROMs shorter and easier to administer and score while retaining accuracy.^30^ Creating computerised adaptive testing versions of PROMs is feasible, with methods outlined elsewhere,^30^ and can be done by researchers/clinicians with familiarity of psychometrics.

Strengths of this study include: the inclusion of a large sample of TGD patients and HCPs diverse in age and gender identity. This study sample represented participants from an English NHS gender clinic. Future research should seek to investigate perspectives from outside the UK; however, this study does have representativeness of a diverse range of gender identities. Further, we used established theories in implementation science to guide the research design and in interpretation of results.^31^ Limitations of this study include a lack of racial and ethnic diversity. Future studies should aim to include the feedback of racial and ethnic minority populations on feedback for the PG-PROM-GAC. Second, there was a high rate of non-respondents for this study, and future research should seek to improve sampling to potentially increase response rates. It is possible that the non-respondents may be more reluctant to accept PROM implementation. This study did not analyse the demographic information of non-respondents as they did not provide this information/consent for it to be used in this way. Future research should seek to investigate demographic information of non-respondents to investigate the full extent that study participants represent diverse perspectives.

## Conclusion

The key reasons that the PG-PROM-GAC is considered acceptable and feasible are feedback from gender-affirming care patients and HCPs mentioning support for the PG-PROM-GAC as a resource that is needed, welcomed and thoughtfully constructed. Participants also felt the PG-PROM-GAC was comprehensive and covered various aspects of relevance for gender-affirming care PROM implementation. Future work with the PG-PROM-GAC should consider its real-world clinical application and use alongside a PROM for gender-affirming care.

## Data Availability

Data are available upon reasonable request. Requests for data can be made to the corresponding author.
